# Evaluating the role of time in range as a glycemic target during short‐term intensive insulin therapy in patients with newly diagnosed type 2 diabetes

**DOI:** 10.1111/1753-0407.13355

**Published:** 2023-01-17

**Authors:** Liehua Liu, Weijian Ke, Lijuan Xu, Hai Li, Juan Liu, Xuesi Wan, Jianbin Liu, Wanping Deng, Xiaopei Cao, Haipeng Xiao, Yanbing Li

**Affiliations:** ^1^ Department of Endocrinology the First Affiliated Hospital of Sun Yat‐Sen University Guangzhou China; ^2^ Endocrinology Department Eastern Health Melbourne Victoria Australia

**Keywords:** newly diagnosed, remission, short‐term intensive insulin therapy, time in range, type 2 diabetes, 初诊, 缓解, 短期胰岛素强化治疗, 葡萄糖在目标范围内时间, 2型糖尿病。

## Abstract

**Background:**

Tight glycemic control during short‐term intensive insulin therapy (SIIT) is critical for inducing diabetes remission in patients with newly diagnosed type 2 diabetes (T2D). This work aimed to investigate the role of time in range (TIR) during SIIT as a novel glycemic target by predicting clinical outcomes.

**Methods:**

SIIT was given to 116 patients with newly diagnosed T2D, with daily eight‐point capillary glucose monitored. Glycemic targets (fasting/premeal glucose, 3.9–6.0 mmol/L; 2 h postprandial blood glucose, 3.9–7.8 mmol/L) were achieved and maintained for 2 weeks. TIR_PIR_ was calculated as the percentage of glucose points within these glycemic targets during the maintenance period and was compared to TIR_3.9–7.8mmol/L_ and TIR_3.9–10.0mmol/L_. Acute insulin response (AIR), HOMA‐IR, HOMA‐B, and disposition index (DI) were measured. Patients were followed up for 1 year to observe clinical outcomes.

**Results:**

TIR_PIR_, TIR_3.9–7.8mmol/L_, and TIR_3.9–10.0mmol/L_ were 67.2 ± 11.2%, 80.8 ± 9.2%, and 90.1 ± 6.2%, respectively. After SIIT, β‐cell function and insulin sensitivity improved remarkably, and the 1‐year remission rate was 55.2%. △AIR and △DI were positively correlated with all the TIR values, whereas only TIR_PIR_ was correlated with △HOMA‐IR (r = −0.22, *p* = 0.03). Higher TIR_PIR_ but not TIR_3.9–7.8mmol/L_ or TIR_3.9–10.0mmol/L_ was robustly associated with diabetes remission; patients in the lower TIR_PIR_ tertile had an elevated risk of hyperglycemia relapse (hazard ratio 3.4, 95% confidence interval 1.6–7.2， *p* = .001). Only those with TIR_PIR_ ≥ 65% had a one‐year remission rate of over 60%.

**Conclusions:**

These findings advocate TIR_PIR_ ≥ 65% as a novel glycemic target during SIIT for clinical decision‐making.

## Graphical abstract

**Highlights**

- Whether time in range (TIR) during short‐term intensive insulin therapy (SIIT) was associated with treatment response to the therapy in patients with newly diagnosed type 2 diabetes was uncertain.
- TIR_PIR_, which was calculated according to the percentage of glucose points within predefined glycemic targets, was superior to TIR_3.9–7.8mmol/L_, TIR_3.9–10.0mmol/L_, and other glycemic markers in predicting recovery of β‐cell function, improvement of insulin sensitivity, and 1‐year remission.
- Patients with TIR_PIR_ ≥ 65% had a 1‐year remission rate of 60% or higher.

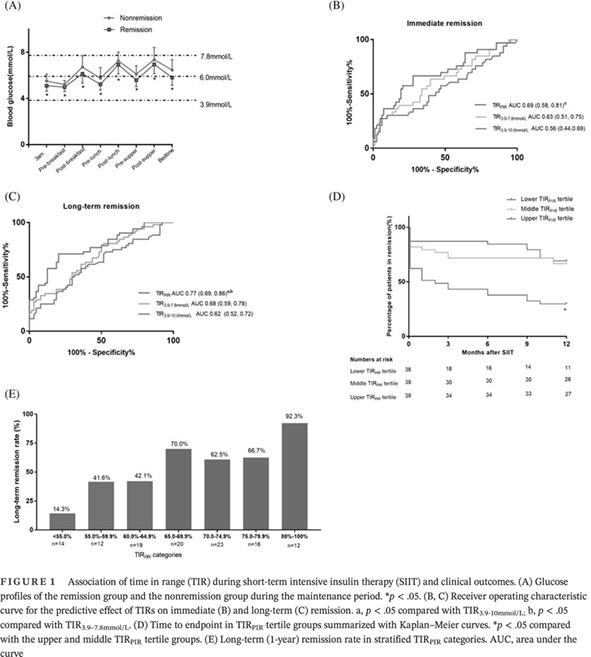

## 摘要

**背景:**短期强化胰岛素治疗(SIIT)期间的严格血糖控制对新诊断2型糖尿病(T2D)患者的糖尿病缓解至关重要。本研究旨在探讨SIIT期间葡萄糖目标范围内时间(TIR)作为预测临床结局的新型血糖目标的作用。

**方法:**对116例新诊断的T2D患者进行SIIT治疗, 每日监测8‐点毛细血管血糖。血糖控制达标(空腹/餐前血糖, 3.9~6.0 mmol/L;餐后2h血糖3.9 ~ 7.8mmol/L), 并维持2周。TIR_PIR_计算为维持期这些血糖目标内的血糖点百分比, 并与TIR_3.9‐7.8mmol/L_和TIR_3.9‐10.0mmol/L_进行比较。测定急性胰岛素反应(AIR)、(HOMA‐IR、HOMA‐B和葡萄糖处置指数(DI)。随访患者1年, 观察临床疗效。

**结果:**TIR_PIR_、TIR_3.9 ~ 7.8mmol/L_和TIR_3.9 ~ 10.0mmol/L_分别为67.2±11.2%、80.8±9.2%和90.1±6.2%。SIIT后患者胰岛β细胞功能及胰岛素敏感性明显改善, 1年缓解率为55.2%。△AIR、△DI与所有TIR值均呈正相关, 仅TIR_PIR_与△HOMA‐IR呈正相关(r=‐0.22, P=0.03)。较高的TIR_PIR_与糖尿病缓解密切相关, 而TIR_3.9‐7.8mmol/L_或TIR_3.9‐10.0mmol/L_与糖尿病缓解无关, 低TIR_PIR_三分位组患者高血糖复发风险增加(HR 3.4, 95%CI 1.6 ~ 7.2, P=0.001)。只有TIR_PIR_≥65%的患者1年缓解率超过60%。

**结论:**建议将TIR_PIR_≥65%作为SIIT期间新的血糖控制目标, 以指导临床决策。

## 1 INTRODUCTION

Diabetes affects as many as 537 million people globally and 12.4% of adults in China. More than half of the cases are newly diagnosed.^1^, ^2^ In the highly impactful UK Prospective Diabetes Study, β‐cell function had reduced by 50% and continued to decline in type 2 diabetes (T2D),^3^ leading to progressively worsening of blood glucose control. Based on this understanding, T2D was considered a lifelong progressive disease. An escalation glucose‐lowering strategy, that is, increasing numbers of antidiabetic agents as blood glucose control deteriorates, was well accepted. However, glycemic control remained suboptimal in more than 50% of the patients for decades.^4^

In recent years, reversing T2D was found possible by applying aggressive managements such as weight loss,^5^ bariatric surgery,^6^ and short‐term intensive insulin therapy (SIIT).^7^ The reversibility of T2D attracted much attention because the benefit of glycemic control had a “legacy” effect.^8^ The importance of early and sustained near‐normoglycemia was confirmed in a large cohort study, which showed that even exposure to mild hyperglycemia (glycated hemoglobin A1c [HbA1c] ≥ 6.5%) during the first year after diagnosis was associated with a higher incidence of microvascular and macrovascular increase worse outcomes.^9^

SIIT was introduced into the management of early T2D in the past 2 decades since its reversal effect was validated by studies from our center and all around the world. The therapy induces amelioration of β‐cell dysfunction and drug‐free diabetes remission for over 1 year in half of the patients with newly diagnosed T2D.^7^, ^10^, ^11^, ^12^, ^13^ Based on this evidence, SIIT has now been recommended by the guideline from the Chinese Diabetes Society as standard therapy for patients with newly diagnosed T2D who have remarkable hyperglycemia.^14^ The elimination of glucotoxicity was considered to play a critical role in obtaining favorable treatment response. In previous studies,^7^, ^11^, ^12^ glycemic targets during SIIT were stringent (fasting blood glucose [FBG]<6.1 mmol/L, 2 h postprandial glucose [2hPG] < 8.0 mmol/L). In our previous study, patients with lower daily mean blood glucose during the maintenance period of SIIT had better improvement in β‐cell function and a higher chance of having long‐term remission.^15^ Thus, SIIT should be carefully executed with glycemic parameters monitored so that insulin infusion can be better titrated to achieve near‐normoglycemic targets.

However, there are still barriers that affect the implementation of SIIT. One of the unmet requirements was the standardization and optimization of the therapy. For example, the lack of markers precisely predicting treatment response makes the clinical decision about subsequent management after SIIT unclear for the physician. Moreover, the current glycemic targets that were set for insulin dosing during the therapy were mainly based on experience. To what extent will the glucose fluctuation during SIIT affect the treatment response is largely uncertain. Likewise, it would be helpful to introduce new glycemic markers to facilitate the execution of the therapy. Although lower daily mean blood glucose during the maintenance period of SIIT was associated with better glycemic outcomes,^15^ the marker has clear limitations as a glycemic target because of its retrospective property and lack of association with glucose excursion.

Recently, time in range (TIR), together with time above range (TAR) and time below range (TBR), were recommended as novel metrics of glycemic control.^16^ Unlike HbA1c, these indices represented both overall glycemic control and glucose fluctuation. In cohort studies, TIR was shown to have a significant association with the development of chronic diabetic complications such as retinopathy and diabetic kidney disease^17^, ^18^ and even all‐cause and cardiovascular mortality.^19^ It was of interest to investigate if TIR during SIIT is valuable in predicting clinical outcomes concerning reversal of diabetes, and if so, what are the clinical implications for SIIT practice. In addition, there are different calculation manners of TIR. When derived from continuous glucose monitoring data, TIR refers to the time an individual spends within a specified target glucose range (usually 3.9–10.0 mmol/L, sometimes 3.9–7.8 mmol/L).^16^ In the literature, TIR derived from capillary glucose monitoring can also be calculated as the proportion of glucose values within predefined ranges; sometimes, it was expressed as the form of points in range (PIR).^20^, ^21^ Which calculation was more suitable for generating TIR in SIIT required exploration. Therefore, we conducted this study investigating the association of TIR values calculated using different methods with treatment response to evaluate the potential role of TIR as a novel glycemic target for SIIT execution.

## 2 METHODS

### 2.1 Participants

The patients' data were collected from two independent randomized controlled trials (NCT00948324 and NCT01471808) conducted in The First Affiliated Hospital of Sun Yat‐sen University with a similar study design. NCT00948324 was designed to evaluate whether SIIT combined with metformin, rosiglitazone, or α‐lipoic acid was superior to SIIT alone in terms of long‐term glycemic control.^22^ NCT01471808 was done to compare the effects of SIIT plus different add‐on therapy (pioglitazone+ metformin or sitagliptin) to SIIT alone on diabetes remission. Only data from SIIT alone groups from the two trials were pooled. The enrolled patients were newly diagnosed with T2D according to the World Health Organization (1999) criteria^23^ without any previous usage of antihyperglycemic agents. Other inclusion criteria included 25 and 70 years of age, body mass index (BMI) between 21 and 35 kg/m^2^ and FPG between 7.0 and 16.7 mmol/L. Patients were excluded if they had acute or severe chronic diabetic complications, severe concomitant abnormalities, or usage of medicines that markedly influence blood glucose. This research was approved by the research ethics board of Sun Yat‐Sen University, with signed informed consent obtained from each patient.

### 2.2 Study design

The protocol of SIIT and subsequent follow‐up were identical in the two trials and had been described elsewhere.^15^, ^22^ In brief, all patients were admitted to the hospital and received SIIT delivered using an insulin pump. The total daily insulin dose was initiated by 0.5 IU/kg, divided 50:50 into basal insulin and premeal boluses. The insulin infusion regimen was titrated according to eight‐point capillary blood glucose values (before and 2 h after three meals, at bedtime, and at 3 am) with fasting/premeal blood glucose<6.1 mmol/L and 2hPG <8.0 mmol/L as the targets. Once the glycemic targets were achieved, insulin infusion was maintained for 14 days (maintenance period). During hospitalization, the diet was provided according to the recommendation of nutritionists. Carbohydrates, proteins, and fat accounted for 50%–60%, 10%–15%, and 20%–30% of total energy intake. Patients were requested to take a walk or jog for 30–60 min after each meal. After insulin was suspended, patients were followed up for up to 1 year with blood glucose monitored. Diabetes remission was defined as FBG <7.0 mmol/L and 2hPG < 10.0 mmol/L without hypoglycemic agents. Diabetes remission achieved immediately after SIIT and over 1 year were defined as immediate remission and long‐term remission, respectively. Once hyperglycemia relapse was confirmed, antihyperglycemic treatment was initiated following the current Chinese guideline.^14^

### 2.3 Blood sampling and measurements

At baseline and after SIIT, anthropometric data such as body weight, height, and waist circumference were recorded. Venous blood was collected for measurements of FPG, 2hPG, serum fasting lipid profiles, and liver enzymes. An intravenous glucose tolerance test (IVGTT) was performed the following day by administering 50 ml of 50% glucose, with serum samples obtained before and 1, 2, 4, 6, and 10 min after glucose load for insulin assay.^7^ Acute insulin response (AIR) was calculated as the incremental trapezoidal area during the IVGTT. Homeostasis model assessment was applied to estimate β‐cell function (HOMA‐β) and insulin resistance (HOMA‐IR). The disposition index (DI) was calculated by AIR×1/HOMA‐IR (HOMA‐S).^24^ All laboratory tests were done in the Central Clinical Lab of the First Affiliated Hospital of Sun Yat‐Sen University.

TIR was generated using the eight‐point capillary blood glucose values with three different methods: (1) TIR_PIR_: the percentage of time that glucose values within the glycemic targets (for nocturnal, fasting or premeal blood glucose, 3.9–6.0 mmol/L; for 2hPG, 3.9–7.8 mmol/L); 2, TIR_3.9–7.8mmol/L_: the percentage of time that glucose values were between 3.9–7.8 mmol/L; 3. TIR_3.9–10.0mmol/L_: the percentage of time that glucose values were between 3.9–10.0 mmol/L. The corresponding TARs and TBRs, daily mean blood glucose and glucose variability (evaluated by SD [SDBG] and coefficient of variation [CV] of blood glucose) were also calculated.

### 2.4 Statistical analysis

Normally distributed data were presented as mean ± SD and compared using one‐way analysis of variance or Student's *t* tests (paired *t* tests for comparisons before and after SIIT, unpaired *t* tests for comparisons between independent groups). Nonnormally distributed data were presented as median (interquartile range) and compared with the Kruskal–Wallis H tests or Mann–Whitney tests. The associations of variables were assessed with Pearson correlation or Spearman correlation. Proportions were compared with the chi‐square test. Logistic regression was applied to determine predictors of remission. Receiver operating characteristic curve (ROC) analyses were used to compare the predictive effect of TIRs. Time‐to‐event distributions were summarized with the Kaplan–Meier curve, with risk factors estimated using Cox proportional‐hazards models. All statistical procedures were accomplished with SPSS version 19.0 and Graphpad Prism version 7.0.

## 3 RESULTS

### 3.1 The reversal of T2D induced by SIIT

**FIGURE 1:**
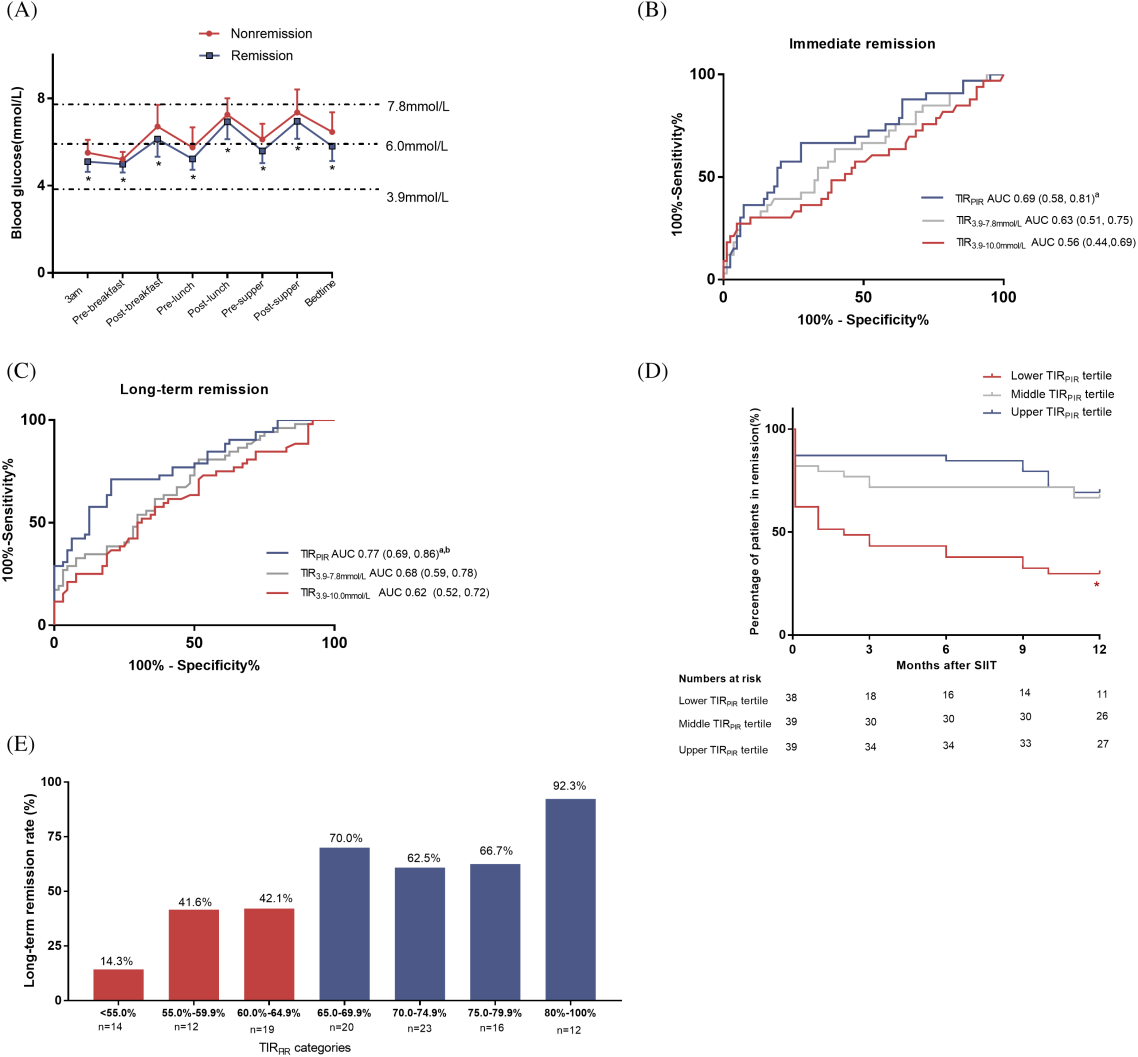
Association of time in range (TIR) during short‐term intensive insulin therapy (SIIT) and clinical outcomes. (A) Glucose profiles of the remission group and the nonremission group during the maintenance period. *p < .05. (B, C) Receiver operating characteristic curve for the predictive effect of TIRs on immediate (B) and long‐term (C) remission. a, *p* < .05 compared with TIR_3.9‐10mmol/L;_ b, *p* < .05 compared with TIR_3.9–7.8mmol/L_. (D) Time to endpoint in TIR_PIR_ tertile groups summarized with Kaplan–Meier curves. *p < .05 compared with the upper and middle TIR_PIR_ tertile groups. (E) Long‐term (1‐year) remission rate in stratified TIR_PIR_ categories. AUC, area under the curve

There were 40 patients and 93 patients with newly diagnosed T2D in the SIIT group from NCT00948324 and NCT01471808, respectively. Seventeen patients were excluded because of a lack of follow‐up data. Thus, the remaining 116 patients were enrolled. The patients had a mean age of 48.1 ± 9.4 years, a mean BMI of 25.0 ± 2.9 kg/m^2^, and a mean HbA1c of 11.2 ± 2.2% (99 ± 24 mmol/mol). Glycemic targets were achieved in 3.4 ± 2.0 days. During the maintenance period of SIIT, overall mean blood glucose, SDBG, and glucose CV were 6.0 ± 0.5 mmol/L, 1.7 ± 0.4 mmol/L, and 27.3 ± 6.0% (Figure 1A). TIR_PIR_, TIR_3.9–7.8mmol/L_, and TIR_3.9–10.0mmol/L_ were 67.2 ± 11.2%, 80.8 ± 9.2%, and 90.1 ± 6.2%, respectively.

As shown in Table 1, β‐cell function (measured by HOMA‐β and AIR), insulin sensitivity (measured by HOMA‐IR), and DI were all improved after SIIT. Eighty‐four patients (72.4%) achieved diabetes remission immediately after SIIT (immediate remission). Sixty‐four patients (55.2%) remained in remission status at the end of 1‐year follow‐up. Patients with long‐term diabetes remission (>1 year) had significantly higher TIRs, lower TARs, lower mean blood glucose, and less glucose variability during SIIT. Better recovery in β‐cell function and insulin sensitivity after SIIT was also observed in the long‐term remission group.

**TABLE 1:** Effects of SIIT on clinical measurements in patients with newly diagnosed T2D

	Nonremission *N* = 52	Remission *N* = 64	Overall *N* = 116	*p* value
Age (years)	49.9 ± 9.6	46.7 ± 9.1	48.1 ± 9.4	.07
BMI (kg/m^2^)				
Before SIIT	24.8 ± 3.1	25.2 ± 2.8	25.0 ± 2.9	.41
After SIIT	24.6 ± 3.2*	24.9 ± 2.7*	24.8 ± 2.9*	.57
HbA1c				
Before SIIT	11.5 ± 2.2% (102 ± 24 mmol/mol)	10.9 ± 2.2% (96 ± 24 mmol/mol)	11.2 ± 2.2% (99 ± 24 mmol/mol)	.19
After SIIT	9.6 ± 1.7% (81 ± 19 mmol/mol)**	9.1 ± 1.6% (76 ± 17 mmol/mol)**	9.3 ± 1.7% (78 ± 19 mmol/mol)**	.10
Alanine aminotransferase (U/L)				
Before SIIT	22.0 (16.2, 33.0)	24.0 (18.0, 40.0)	24.0 (17.0, 37.3)	.10
After SIIT	21.0 (17.0, 25.5)	24.0 (19.0, 33.0)	22.0 (18.0, 31.0)	.02
Aspartate transferase (U/L)				
Before SIIT	22.0 (16.0，27.8)	24.0 (17.0,36.0)	23.0 (17.0, 32.5)	.11
After SIIT	21.0 (16.0, 27.0)	23.0 (18.0,31.0)	22.0 (17.0, 29.8)	.17
FPG (mmol/L)				
Before SIIT	12.4 ± 2.8	11.3 ± 3.2	11.8 ± 3.1	.062
After SIIT	7.5 ± 2.0**	6.1 ± 0.8**	6.7 ± 1.6**	<.001
2hPG (mmol/L)				
Before SIIT	19.3 ± 5.7	18.2 ± 6.2	18.7 ± 6.0	.33
After SIIT	10.6 ± 3.0*	7.9 ± 2.1**	9.1 ± 2.9**	<.001
AIR (μU·ml^−1^·min)				
Before SIIT	−7.8 (−18.4, 3.1)	−8.9 (−25.9, −2.9)	−8.5 (−24.7, −0.9)	.49
After SIIT	29.6 (16.3, 68.9)**	53.8 (26.0, 106.5)**	47.2 (21.4, 78.2)**	.01
△AIR, (μU·ml^−1^·min)	40.2 (28.3, 75.2)	74.5 (34.4, 121.6)	55.6 (30.0, 94.8)	.002
HOMA‐IR				
Before SIIT	2.7 (1.8,4.5)	3.2 (2.0,4.8)	3.0 (1.9, 4.5)	.46
After SIIT	2.1 (1.4, 2.8)**	1.9 (1.2, 2.8)**	1.9 (1.3, 2.8)**	.23
△HOMA‐IR	−0.7 (−1.9, 0.2)	−1.2 (−2.3, −0.6)	−1.0 (−2.1, −0.2)	.026
HOMA‐β				
Before SIIT	12.2 (6.8, 25.3)	18.7 (9.4,32.3)	17.6 (8.1,27.8)	.01
After SIIT	35.8 (24.2,58.6)**	52.0 (35.1, 71.4)**	42.4 (29.1, 65.2)**	.004
△HOMA‐β	19.3 (8.4,39.5)	28.9 (17.2,43.8)	25.8 (12.6,41.6)	.10
DI				
Before SIIT	−3.3 (−5.4,0.9)	−3.6 (−5.8, −0.8)	−3.4 (−5.8, −0.3)	.48
After SIIT	18.1 (8.2, 39.7)**	29.7 (14.4, 69.5)**	24.7 (11.8, 54.6)**	.009
△DI	20.9 (8.9, 37.7)	35.0 (17.9, 69.9)	28.1 (14.8, 56.1)	.001
Total cholesterol (mmol/L)				
Before SIIT	5.8 ± 1.2	5.5 ± 1.4	5.6 ± 1.3	.17
After SIIT	5.2 ± 1.1**	5.2 ± 1.2**	5.2 ± 1.1**	.87
Triglyceride (mmol/L)				
Before SIIT	1.9 ± 1.1	2.0 ± 1.3	1.9 ± 1.2	.57
After SIIT	1.3 ± 0.6**	1.2 ± 0.4**	1.2 ± 0.5**	.20
HDL‐C (mmol/L)				
Before SIIT	1.2 ± 0.3	1.1 ± 0.3	1.1 ± 0.3	.21
After SIIT	1.2 ± 0.2	1.2 ± 0.2*	1.2 ± 0.2*	.86
LDL‐C (mmol/L)				
Before SIIT	3.9 ± 1.0	3.8 ± 1.2	3.8 ± 1.2	.49
After SIIT	3.5 ± 0.9**	3.5 ± 1.0**	3.5 ± 1.0**	.60
Mean blood glucose (mmol/L)	6.3 ± 0.5	5.8 ± 0.3	6.0 ± 0.5	<.001
SDBG (mmol/L)	1.8 ± 0.4	1.5 ± 0.4	1.7 ± 0.4	.001
Glucose CV (%)	28.4 ± 6.1	26.3 ± 5.9	27.3 ± 6.0	.07
TIR_PIR_ (%)	61.0 ± 11.5	72.2 ± 8.1	67.2 ± 11.2	<.001
TAR_PIR_ (%)	33.7 ± 11.7	22.5 ± 8.3	27.5 ± 11.4	<.001
TBR_PIR_ (%)	5.3 ± 4.5	5.3 ± 4.4	5.3 ± 4.5	.95
TIR_3.9–7.8mmol/L_ (%)	83.4 ± 6.6	77.5 ± 10.7	80.8 ± 9.2	<.001
TAR_3.9–7.8mmol/L_ (%)	11.3 ± 5.4	17.2 ± 9.6	13.9 ± 8.1	<.001
TBR_3.9–7.8mmol/L_ (%)	5.3 ± 4.5	5.3 ± 4.4	5.3 ± 4.5	.95
TIR_3.9–10.0mmol/L_ (%)	92.2 ± 5.1	89.0 ± 7.1	90.1 ± 6.2	.005
TAR_3.9–10.0mmol/L_ (%)	2.9 ± 2.8	5.7 ± 4.8	4.2 ± 4.0	<.001
TBR_3.9–10.0mmol/L_ (%)	5.3 ± 4.5	5.3 ± 4.4	5.3 ± 4.5	.95

Abbreviations: 2hPG, 2‐h postprandial blood glucose; AIR, acute insulin response; BMI, body mass index; CV, coefficient of variance; DI, disposition index; FPG, fasting plasma glucose; HbA1c, glycated hemoglobin A1c; HDL‐C, high‐density lipoprotein cholesterol; HOMA, homeostasis model assessment; LDL‐C, low‐density lipoprotein cholesterol; SDBG, SD of blood glucose; SIIT, short‐term intensive insulin therapy; T2D, type 2 diabetes; TAR, time above range; TBR, time below range; TIR, time in range.

* *p* < .05 compared with before SIIT.

** *p* < .001 compared with before SIIT. Data are mean ± SD or median (interquartile range).

### 3.2 TIR_PIR_ was associated with better clinical outcomes after SIIT

We further compared the predictive effect of TIR values calculated using different principles on clinical outcomes. After adjusting for baseline BMI, age, and FPG, all TIRs were significantly correlated with △AIR (*r* = 0.34, 0.31, and 0.28 for TIR_PIR_, TIR_3.9–7.8mmol/L_ and TIR_3.9–10.0mmol/L_ respectively, *p* = .001, .002, and .005 respectively) and △DI (*r* = 0.34, 0.28, and 0.20 for TIR_PIR,_ TIR_3.9–7.8mmol/L_, and TIR_3.9–10.0mmol/L_ respectively, *p* = .001, .005, and .041 respectively), with the correlation coefficient numerically the largest in TIR_PIR_. Only TIR_PIR_ significantly correlated with △HOMA‐IR (*r* = −0.22, *p* = .03).

ROC curve analyses were further applied to evaluate the predictive effect of TIRs on diabetes remission (Figure 1B,C). For immediate remission, the area under the curve (AUC) of ROC curves for TIR_PIR_, TIR_3.9–7.8mmol/L_, and TIR_3.9–10.0mmol/L_ were 0.69, 0.63, and 0.56, respectively. Comparison between AUC for TIR_PIR_ versus AUC for TIR_3.9–10.0mmol/L_ reached statistical significance (*z* = 2.17, *p* = .03). For long‐term remission, the AUC for ROC curves for TIR_PIR_ was significantly larger than that for TIR_3.9–7.8mmol/L_ (0.77 vs. 0.68, *z* = 2.50, *p* = .012) and TIR_3.9–10.0mmol/L_ (0.77 vs. 0.62, *z* = 3.04, *p* = .002).

We next performed logistic regression analyses to confirm TIR_PIR_ as an independent predictor of long‐term remission. TIR_PIR_ values were analyzed as a binary categorical variable with a cutoff point of the 50th percentile (68.6%). The details of the models are described in Table 2. TIR_PIR,_ TIR_3.9–10.0mmol/L,_ and TIR_3.9–10.0mmol/L_ were selected into the model using a stepwise strategy if the *p* value was less than .1. Other variables were forced into the model for adjustment. Higher TIR_PIR_ significantly increased the chance of long‐term remission (odds ratio 4.52, 95% confidence interval [CI] 2.00–10.21, *p* < .001), adjusting for age, baseline BMI, and HbA1c (model 1). This association was unchanged when baseline HOMA‐β, HOMA‐IR, AIR, and DI were further adjusted (model 2). When glycemic markers during SIIT (mean blood glucose, SDBG, CV of glucose, model 3) or variables after SIIT (FPG and 2hPG after therapy, △HOMA‐β, △HOMA‐IR, △AIR, and △DI, model 4) were further adjusted, this association was attenuated but remained statistically significant. None of the TIR_3.9–7.8mmol/L_ or TIR_3.9–10.0mmol/L_ was able to enter the final models.

**TABLE 2:** Logistic regression on the association of predictors and 1‐year glycemic remission.

Models	Predictors	OR	95% CI	*p* value
Model 1:Adjusting for age, BMI, and baseline HbA1c	TIR_PIR_ (≥68.6%)	4.52	2.0–10.21	<.001
Model 2: Adjusting for variables in model 1 + baseline AIR, HOMA‐IR, HOMA‐β, and disposition index	TIR_PIR_ (≥68.6%)	4.05	1.72–9.5	.001
	HOMA‐β (per unit)	1.05	1.00–1.10	.022
Model 3: Adjusting for variables in model 2+ Mean blood glucose, glucose CV, SDBG	TIR_PIR_ (≥68.6%)	2.77	1.00–7.69	.05
	HOMA‐β (per unit)	1.05	1.00–1.10	.034
Model 4: Adjusting for variables in model 2 + FPG and 2hPG after SIIT, △AIR，△DI，△HOMA‐β, and △HOMA‐IR	TIR_PIR_ (≥68.6%)	3.01	1.11–8.14	.029
	HOMA‐β (per unit)	1.06	0.99–1.14	.08
	2hPG (per mmol/L)	0.79	0.60–1.03	.09

Abbreviations: 2hPG, 2‐h postprandial blood glucose; AIR, acute insulin response; BMI, body mass index; CV, coefficient of variance; DI, disposition index; FPG, fasting plasma glucose; DI, disposition index; HbA1c, glycated hemoglobin A1c; HOMA, homeostasis model assessment；OR, odds ratio; SDBG, SD of blood glucose; SIIT, short‐term intensive insulin therapy; TIR, time in range.

### 3.3 Evaluating TIR_PIR_ as a glycemic target of SIIT

Having identified that TIR_PIR_ was independently associated with long‐term glycemic outcomes, we further explored a suitable cutoff point as an applicable glycemic target during SIIT. Patients were first categorized into three groups by tertiles of TIR_PIR_ (<63.8%, 63.8%–71.7%, and >71.7%). As shown in Table 3, patients with younger age, lower baseline HbA1c and blood glucose, higher liver enzymes, and higher HOMA‐β tended to achieve higher TIR_PIR_ during SIIT. Consistent with the correlation analysis, from the lower tertile group to the upper tertile group, there were stepwise increases in △DI and △AIR and a stepwise decrease in △HOMA‐IR. As shown in Figure 1D, both the upper tertile group (69.3%) and the middle tertile group (66.7%) had higher 1‐year remission rates than that of the lower tertile group (28.9%, *p* < 0.05). In the Cox regression analysis, the lower TIR tertile group had a significantly higher risk of hyperglycemic relapse during follow‐up (hazard ratio 3.4, 95% CI 1.6–7.2, *p* = .001) after adjusting for age, BMI, and baseline HbA1c.

**TABLE 3:** Characteristics of patients in different TIR categories

	Lower TIR_PIR_ tertile	Middle TIR_PIR_ tertile	Upper TIR_PIR_ tertile	*p* value
Age (years)	51.3 ± 9.5^‡^	46.6 ± 10.3^†^	46.6 ± 7.7^†^	.04
BMI (kg/m^2^)				
Before SIIT	24.4 ± 3.2	25.1 ± 2.9	25.6 ± 2.6	.21
After SIIT	24.2 ± 3.2	24.7 ± 2.8	25.4 ± 2.7	.27
HbA1c				
Before SIIT	11.8 ± 2.1% (105 ± 23 mmol/mol)	11.3 ± 2.3% (100 ± 25 mmol/mol)	10.4 ± 1.9% (90 ± 21 mmol/mol)^†^	.01
After SIIT	9.9 ± 1.7% (85 ± 18 mmol/mol)	9.3 ± 1.7% (78 ± 19 mmol/mol)	8.8 ± 1.6% (73 ± 17 mmol/mol)^†^	.02
ALT (U/L)				
Before SIIT	21.0 (15.7, 32.2)	22.0 (16.5, 35.2)	30.5 (21.7, 42.7)^†^ ^,^ ^‡^	.005
After SIIT	21.0 (17.2, 25.0)	22.0 (17.0, 26.2)	28.5 (19.0, 36.2)^†^ ^,^ ^‡^	.009
AST (U/L)				
Before SIIT	21.5 (16.0, 27.0)	22.5 (15.7, 30.5)	26.0 (19.7, 36.2)^†^	.065
After SIIT	22.0 (16.0, 27.7)	20.0 (15.0, 28.0)	24.5 (19.7, 35.2)	.122
FPG (mmol/L)				
Before SIIT	12.8 ± 2.9	11.8 ± 3.3	11.0 ± 2.9^†^	.04
After SIIT	7.7 ± 2.2	6.3 ± 0.8^†^	6.2 ± 1.1^†^	<.001
2hPG (mmol/L)				
Before SIIT	20.3 ± 5.7	18.7 ± 6.0	17.1 ± 6.0^†^	.07
After SIIT	11.0 ± 3.0	8.3 ± 2.4^†^	8.0 ± 2.3^†^	<.001
AIR (μU·ml^−1^·min)				
Before SIIT	−9.7 (−25.4, 0.4)	−9.8 (−18.3, −4.2)	−6.4 (−25.8, 5.6)	.612
After SIIT	24.6 (12.4, 43.0)	46.8 (21.9, 78.3)^†^	69.0 (47.6, 102.4)^†^ ^,^ ^‡^	<.001
HOMA‐IR				
Before SIIT	3.0 (2.2, 4.6)	2.6 (1.8, 4.1)	3.3 (2.0, 4.6)	.765
After SIIT	1.9 (1.5,3.8)	1.8 (1.2, 2.2)^†^	2.0 (1.0, 2.9)	.111
HOMA‐β				
Before SIIT	12.1 (6.9, 22.4)	14.0 (8.3, 29.1)	20.4 (13.2, 28.6)^†^	.078
After SIIT	38.0 (22.2, 54.4)	40.6 (30.3, 67.2)	48.8 (38.7, 70.0)^†^	.092
DI				
Before SIIT	−2.7 (−5.3,0.2)	−3.7 (−5.8, −1.7)	−2.5 (−7.2, 2.1)	.575
After SIIT	11.6 (3.9, 22.8)	29.6 (15.7, 55.5)^†^	40.8 (22.5, 73.6)^†^	<.001
Total cholesterol (mmol/L)				
Before SIIT	5.8 ± 1.3	5.6 ± 1.4	5.4 ± 1.4	.38
After SIIT	5.4 ± 1.1	5.3 ± 1.2	5.0 ± 1.1	.32
Triglyceride (mmol/L)				
Before SIIT	1.9 ± 1.2	1.9 ± 1.2	2.1 ± 1.2	.69
After SIIT	1.3 ± 0.6	1.2 ± 0.4	1.1 ± 0.4	.28
HDL‐C (mmol/L)				
Before SIIT	1.2 ± 0.3	1.1 ± 0.3	1.1 ± 0.3	.78
After SIIT	1.2 ± 0.3	1.2 ± 0.2	1.2 ± 0.2	.76
LDL‐C (mmol/L)				
Before SIIT	3.9 ± 1.0	3.9 ± 1.3	3.8 ± 1.2	.95
After SIIT	3.5 ± 0.9	3.6 ± 1.0	3.4 ± 1.0	.63
Mean blood glucose (mmol/L)	6.4 ± 0.5	6.0 ± 0.3^†^	5.7 ± 0.3^†^ ^,^ ^‡^	<.001
SDBG (mmol/L)	1.9 ± 0.4	1.6 ± 0.4^†^	1.4 ± 0.4^†^ ^,^ ^‡^	<.001
CV of blood glucose	0.3 ± 0.1	0.3 ± 0.1^†^	0.2 ± 0.1^†^ ^,^ ^‡^	<.001
TIR_PIR_%	54.7 ± 8.2	68.2 ± 2.5^†^	78.4 ± 4.9^†^ ^,^ ^‡^	<.001
TAR_PIR_%	39.7 ± 9.4	25.7 ± 4.7^†^	17.5 ± 5.5^†^ ^,^ ^‡^	<.001
TBR_PIR_%	5.6 ± 5.1	6.1 ± 4.0	4.1 ± 4.1	.12
△HOMA‐β	21.2 (8.5, 39.1)	28.7 (14.4, 44.9)	25.8 (12.5, 43.7)	.808
△HOMA‐IR	−0.7 (−1.6, 0.2)	−1.0 (−1.9, −0.3)	−1.3 (−2.9, −0.4)^†^	.097
△DI	14.9 (7.8, 27.6)	34.9 (17.9, 57.4)^†^	38.8 (25.0, 76.6)^†^	<.001
△AIR	34.4 (28.4, 63.7)	50.4 (27.8, 125.7)^†^	79.2 (58.1, 120.2)^†^	<.001

*Note*: Data are mean ± SD or median (interquartile range).

Abbreviations: 2hPG, 2‐hour postprandial blood glucose; AIR, acute insulin response; ALT, alanine transaminase; AST, aspartate transaminase; BMI, body mass index; CV, coefficient of variance; FPG, fasting plasma glucose; DI, disposition index; HbA1c, glycated hemoglobin A1c; HDL‐C, high‐density lipoprotein cholesterol; HOMA, homeostasis model assessment; LDL‐C, low‐density lipoprotein cholesterol; SDBG, SD of blood glucose; SIIT, short‐term intensive insulin therapy；TIR, time in range.

^†^ *p* < .05 compared with lower tertile group.

^‡^ *p* < .05 compared with middle tertile group.

To determine a more precise cutoff point of the TIR_PIR_ target, we further stratified patients into different TIR_PIR_ groups. As shown in Figure 1E, only 14.3%(2/14) of the patients with TIR < 55% obtained long‐term remission. A long‐term remission rate of over 60% was observed only in groups with TIR ≥ 65%. Almost all (11/12, 92.3%) patients with TIR ≥80% achieved 1‐year remission.

## 4 DISCUSSION

In this study, TIR during the maintenance period of SIIT was significantly associated with the recovery of β‐cell function and insulin sensitivity, which led to the reversal of the disease in patients with newly diagnosed T2D. Interestingly, TIR_PIR_, rather than widely recommended TIR_3.9–7.8mmol/L_ and TIR_3.9–10.0mmol/L_, independently predicted diabetes remission. In order to obtain a favorable outcome, a TIR_PIR_ ≥ 65% may be necessary. These results confirmed the importance of near‐normoglycemia during SIIT for reversing T2D and strongly supported considering TIR_PIR_ ≥ 65% as a novel glycemic target for SIIT.

TIR has been suggested as a new glycemic marker because TIR is closely correlated with HbA1c and reflexes blood glucose fluctuation, which partly overcomes some limitations of HbA1c.^25^ TIR has been linked to chronic diabetic outcomes such as microvascular complications (diabetic retinopathy, neuropathy, and nephropathy),^17^, ^18^, ^26^ carotid intima‐media lesions,^27^ and even all‐cause mortality.^19^ Recent analysis also showed that TIR > 80% in critically ill patients treated with intravenous insulin was associated with lower mortality.^28^ To our knowledge, no former study has investigated the impact of TIR during SIIT on the treatment effect of reversing T2D.

Originally, TIR referred to the time spent in a predefined range regardless of time. However, in this study, TIR_PIR_ was calculated using a stricter reference range by considering fasting/premeal status and postprandial status separately. The reason for introducing the new calculation method was based on the acknowledgment of the crucial role of normalizing blood glucose in inducing diabetes remission and β‐cell recovery by SIIT. Loss of functional β‐cell mass in T2D can be attributed to transient or permanent pathophysiological changes such as endoplasmic reticulum stress, oxidative stress, apoptosis, and dedifferentiation.^29^, ^30^ These changes occur as a result of metabolic overload or overstimulation on β cells. In diabetic animal models, reduction of endogenous insulin demand by applying insulin replacement resulted in amelioration of endoplasmic reticulum stress and, more important, triggering β‐cell redifferentiation and enhancing insulin secretion capacity.^31^, ^32^, ^33^ Of note, A slight elevation of blood glucose is sufficient to cause β‐cell dysfunction and dedifferentiation in humans^34^ and rat models.^35^ AIR, a sensitive marker of β‐cell function, remarkably decreased in prediabetes and almost disappeared in overt diabetes.^36^ In our previous study, lower mean blood glucose was associated with better AIR recovery and diabetes remission.^15^ Likewise, in the current study, TIR_PIR_ was also positively correlated with the recovery of AIR and DI. These data suggested that suppressing endogenous insulin secretion via stringently normalizing blood glucose might be essential for impaired β cells to adequately recover from metabolic distress (β‐cell rest effect). Moreover, in previous studies, baseline insulin resistance and recovery of insulin sensitivity after SIIT were also considered vital factors for glycemic remission.^10^ When calculating TIR_3.9–7.8mmol/L_ and TIR_3.9–10.0mmol/L_, a fasting/premeal blood glucose slightly below 7.8 mmol/L (for example, 7.5 mmol/L) is considered within the range. However, this value is not optimal for inducing the β‐cell rest effect and may even be harmful.^34^, ^35^ Taken together, these data explained the finding in our study that TIR_PIR_ was superior to TIR_3.9–7.8mmol/L_ and TIR_3.9–10.0mmol/L_ in predicting treatment outcomes such as diabetes remission, recovery of β‐cell function, and insulin sensitivity, which rationalized the stricter criteria for calculating TIR_PIR_.

The property of TIR makes it potentially valuable in the standardization of treatment. First, given the close relationship between TIR_PIR_ and glycemic outcomes, it is clinically feasible to incorporate it into the SIIT therapeutic strategy as a checkpoint before subsequent treatment can be made. According to the current study, a TIR_PIR_ ≥ 65% (namely, on average more than five points out of the daily eight‐point capillary glucose values are within the predefined targets) indicates a ≥ 60% chance to have long‐term remission; patients with TIR_PIR_ ≥ 80% are even more likely to have long‐term remission. In this case, hypoglycemic agents are likely unnecessary. In contrast, patients with only four or fewer blood glucose points within the targets (TIR_PIR_ ≤ 55%) may have little chance of remission. Hence, sequential antihyperglycemic treatment should be initiated without delay.

Second, TIR_PIR_ provides a novel measure of daily glycemic control quality during SIIT. Although efforts were made to maintain near‐normoglycemia, glycemic excursions may still occur because of insufficient adherence to carbohydrate intake or exercise advice, hypoglycemia, and subsequent therapy modification and correction, etc.^15^ The significance of the off‐target blood glucose was previously unclear because a link to the clinical outcomes was not established. TIR_PIR_ captured information on glycemic excursions and was superior to other glycemic markers such as mean blood glucose, SDBG, and CV of blood glucose in terms of indicating glycemic outcomes, as shown in the predictive models in the current study. More importantly, compared with mean blood glucose, TIR_PIR_ targets are more intuitional for physicians to understand; thus, daily monitoring of TIR may be more practically feasible for lifestyle intervention and fine adjustment of insulin infusion.

There are some limitations of the current study. First, the study cohort was enrolled from a single center. Whether the results can be generalized to different regions or ethnicities requires validation. Second, owing to the design of the original clinical trials, we did not perform continuous glucose monitoring (CGM) to detect dynamic glycemic changes. How the TIR calculated from capillary blood glucose reflects glycemic profiles obtained using CGM and whether glycemic parameters of CGM provide additional information on outcome prediction should be investigated in the future. Moreover, although all participants received standardized recommendations about lifestyle modification from the study staff, self‐management and adherence to lifestyle modification were not evaluated in detail.

In summary, time in range during SIIT was associated with the reversal of hyperglycemia and favorable clinical outcomes in patients with newly diagnosed T2D. TIR_PIR_ ≥ 65% could be incorporated into the standardized management in SIIT as a novel glycemic marker. These findings will throw light on future studies investigating the application of CGM markers in the scenario of SIIT.

## AUTHOR CONTRIBUTIONS

Yanbing Li and Liehua Liu contributed to the conception and study design. Liehua Liu, Weijian Ke, Xuesi Wan, Hai Li, Lijuan Xu, Juan Liu, and Wanping Deng contributed to data acquisition. Yanbing Li, Liehua Liu, and Lijuan Xu contributed to data analysis, interpretation, and drafting. Jianbin Liu, Xiaopei Cao, and Haipeng Xiao verified the data and contributed to critical revision of the work for important intellectual content. Liehua Liu, Weijian Ke, and Lijuan Xu contributed equally to the study. All authors approved the submission of the manuscript.

## CONFLICT OF INTEREST

The authors declare that they have no known competing financial interests or personal relationships that could have appeared to influence the work reported in this paper.

## ETHICS STATEMENT

This research was approved by the research ethics board of Sun Yat‐Sen University.

## PATIENT CONSENT

Signed informed consent obtained from each patient.

## PERMISSION TO REPRODUCE MATERIAL FROM OTHER SOURCES

There is no material from other sources.

## Data Availability

Data will be shared only with bona fide researchers submitting a research proposal and requesting access to data to the corresponding author's email.
